# Evaluation of a Home Monitoring Application for Follow Up after Lung Transplantation—A Pilot Study

**DOI:** 10.3390/jpm10040240

**Published:** 2020-11-21

**Authors:** Nynke Wijbenga, Rogier A. S. Hoek, Bas J. Mathot, Leonard Seghers, Jan J. van Weezel, José den Ouden, Marlies S. Wijsenbeek, Joachim G. J. V. Aerts, Merel E. Hellemons, Catharina C. Moor

**Affiliations:** Department of Pulmonary Medicine, Erasmus Medical Center Rotterdam, 3015 GD Rotterdam, The Netherlands; n.wijbenga@erasmusmc.nl (N.W.); r.hoek@erasmusmc.nl (R.A.S.H.); b.mathot@erasmusmc.nl (B.J.M.); l.seghers@erasmusmc.nl (L.S.); j.vanweezel@erasmusmc.nl (J.J.v.W.); j.denouden@erasmusmc.nl (J.d.O.); m.wijsenbeek-lourens@erasmusmc.nl (M.S.W.); j.aerts@erasmusmc.nl (J.G.J.V.A.); c.moor@erasmusmc.nl (C.C.M.)

**Keywords:** lung transplantation, eHealth, home monitoring, home spirometry

## Abstract

Home spirometry after lung transplantation is common practice, to monitor graft function. However, there is little experience with online home monitoring applications with direct data transfer to the hospital. We evaluated the feasibility and patient experiences with a new online home monitoring application, integrated with a Bluetooth-enabled spirometer and real-time data transfer. Consecutive lung transplant recipients were asked to evaluate this home monitoring application for three months in a pilot study. Home spirometry measurements were compared with in-hospital lung function tests (the forced expiratory volume in 1 s (FEV1) and forced vital capacity (FVC)) at the end of the study. Ten patients participated. The home and hospital spirometry measurements showed a high correlation, for both the FEV1 (*r* = 0.99, *p* < 0.01) and FVC (*r* = 0.99, *p* < 0.01). The adherence and patient satisfaction were high, and the patients preferred the home monitoring application over the current home spirometer, with a difference of 1.4 ± 1.5 points on a scale from 0 to 10 (*p* = 0.02). Online home monitoring with direct data transfer is feasible and reliable after lung transplantation and results in high patient satisfaction. Whether the implementation of online home monitoring enables the earlier detection of lung function decline and improves patient and graft outcomes will be the subject of future research.

## 1. Introduction

Lung transplantation is a lifesaving treatment option in selected patients with end-stage lung disease. Compared to recipients of other solid organs, lung transplant recipients encounter higher health resource utilization, more transplant-related complications, and higher mortality rates [1]. Lung function decline is the first sign of allograft dysfunction from any cause (for example, acute rejection, infection, or chronic rejection), usually before any symptoms arise [2]. The early detection of lung function decline is therefore of utmost importance to ensure early diagnosis and intervention, and is known to reduce later complications, such as chronic allograft dysfunction, and prolong survival [2,3,4].

Currently, all patients in our center perform daily home spirometry on a handheld spirometer and report their results in a diary, which is discussed in the outpatient clinic. Patients are instructed to contact the transplant team if their lung function declines or symptoms arise in between clinic visits; there is no direct data transfer and feedback on changes in lung function and the quality of the measurements outside the hospital, and the monitoring of adherence is not possible. This leads to variability in adherence and responses to changes, and may cause uncertainty in a subgroup of patients in when to contact the hospital. It could be hypothesized that direct data transfer to the lung transplantation center can lead to the earlier detection of non-adherence or a decline in lung function, facilitate personalized treatment, and reduce anxiety in patients.

There is some experience with digital home spirometry applications after lung transplantation, especially with applications giving advice about whether to contact the lung transplant center, but in none of the studies were data transferred directly to the hospital [5,6,7,8]. In this pilot study, we aimed to evaluate the feasibility, reliability, and patient experiences with a new online home monitoring application, integrated with a Bluetooth-enabled spirometer and real-time transfer of data.

## 2. Materials and Methods

### 2.1. Study Design

A prospective pilot study was conducted at the Erasmus Medical Center between June and November 2019. Consecutive lung transplant recipients, irrespective of transplant date, at the outpatient clinic were asked to participate. Because of the non-interventional design, the study was exempt from ethics approval. The patients provided online informed consente.

Patients were invited to evaluate an online home monitoring application (Spirogram), integrated with a Bluetooth-enabled handheld spirometer (MIR Spirobank Smart, Rome, Italy), for three months. The Spirobank Smart Spirometer (Figure S1) is a CE-marked medical device compliant with all safety regulations. Spirogram is a CE-marked eHealth application (Curavista bv, Geertruidenberg, The Netherlands) that can be installed on a smartphone or tablet, and was previously developed together with pulmonary fibrosis patients [9,10].

The patients were asked to perform home spirometry at least once weekly using the home monitoring application, instead of their regular home spirometer (Pulmolife, CareFusion, San Diego, CA, United States of America), in combination with a paper diary. All patients were instructed to perform home spirometry at approximately the same time, to enhance compliance and reduce variability [11]. The forced expiratory volume in 1 s (FEV1) and forced vital capacity (FVC) were transmitted in real time via an encrypted connection, allowing patients and healthcare providers to access these data directly (see Figure S2). The data were stored on a secured server (Gezondheidsmeter, Geertruidenberg, the Netherlands), which has the highest European Certification for safety (NEN7510) and is compliant with all safety regulations (General Data Protection Regulation). In this pilot study, automated alerts for lung function decline had not yet been implemented, but lung function data were reviewed twice weekly by the researchers. Furthermore, the patients were instructed to contact the hospital in the case of lung function decline or symptoms, in line with current practice.

At baseline, the patients received 30 min of training on the use of the spirometer and online application. Patients were considered accurately trained when they were able to perform three good, reproducible FVC measurements (the difference between the two highest FVCs was < 150 mL). A helpdesk was available for all technical questions.

In-hospital measurements of FEV1 and FVC were performed during regular follow-up visits to the outpatient clinic. Patient experiences and Hospital Anxiety and Depression Scale (HADS) scores were recorded at baseline and at the end of the study.

### 2.2. Study Outcomes

To assess the feasibility and reliability of the online home monitoring application, the home spirometry measurements were compared with the in-hospital lung function tests at the end of the study. To do so, the mean of the last three home spirometry measurements was used. Furthermore, we evaluated the within-subject reproducibility of the FEV1 and FVC and adherence to home spirometry.

To compare the patient experiences between both systems, the patients completed an evaluation questionnaire about the PulmoLife spirometer at baseline, and about the home monitoring application and SpiroBank Smart Spirometer at the end of the study. The patients scored the spirometers and application on a visual analogue scale (VAS) from 0 (not pleasant at all/not easy to use) to 10 (very pleasant/very easy to use). The degree of uncertainty about lung function was scored on a scale from 0 (not uncertain at all) to 10 (very uncertain). To assess the impact of the online home monitoring application on anxiety and depression levels, the HADS was completed at baseline and the end of the study [12]. The HADS consists of 14 questions about depression and anxiety. The scores for both domains range from 0 to 21. A score ≥8 indicates that anxiety or depressive symptoms are present.

### 2.3. Statistical Analysis

The home and hospital spirometry measurements were compared with the Pearson correlation coefficient. The within-subject reproducibility was assessed using the coefficient of variation, in patients with stable lung function. Patients with variable measurements in the first weeks received follow-up training. Only measurements after the follow-up training were used for analysis.

The adherence to home spirometry was calculated by dividing the number of weeks in which at least one measurement was performed by the number of weeks in which a patient was able to perform measurements.

A paired *t*-test was used to compare the evaluation questionnaires and HADS at baseline and the end of the study.

The data were analyzed with SPSS statistics (IBM SPSS Statistics for Windows, version 25.0, IBM Corp., Armonk, NY, USA) and are presented as mean ± SD or median (range).

## 3. Results

### 3.1. Baseline Characteristics

Ten patients were included; 50% were male. The mean age was 67 (range, 58–78) years; 90% underwent bilateral transplantation. The underlying disease was chronic obstructive pulmonary disease in three patients, emphysema-related alfa-1-antrypsin deficiency in four patients, and interstitial lung disease in three patients. The patients were, on average, 8 years on from lung transplantation (range, 0.4–19 years). Baseline characteristics can be found in Table 1. Nine out of the ten patients completed the study. The health statuses of these nine patients remained stable during the study according to the treating physician. One patient discontinued the study due to hospital admission after four weeks, because of pneumosepsis.

### 3.2. Feasibility and Reliability

**Figure 1:**
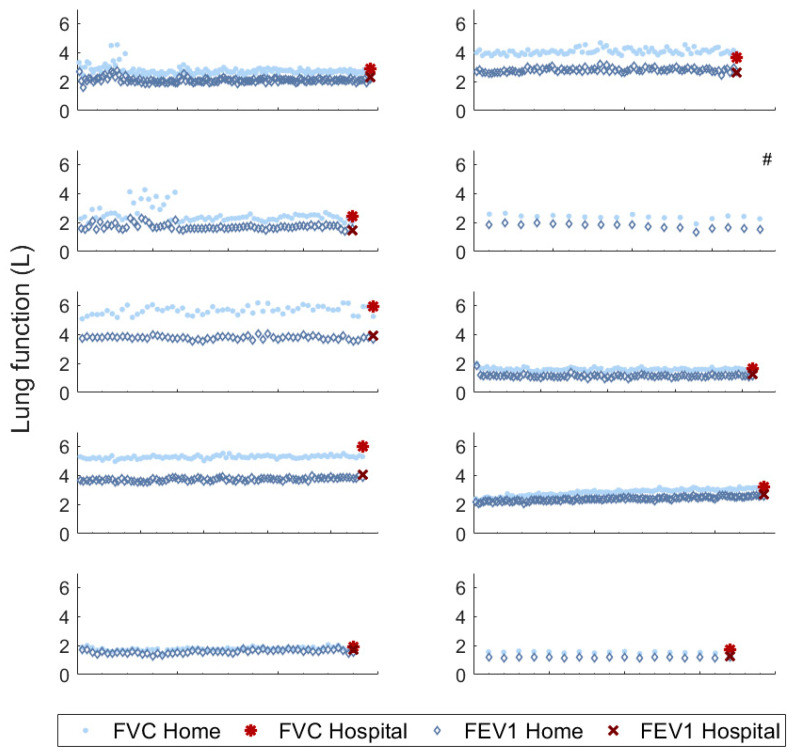
Home and hospital spirometry for all subjects during the study. #: patient did not finish the study due to hospitalization. X-axis represents individual spirometry measurements. Median follow-up in the study was 93 (IQR, 76–130) days. FVC: forced vital capacity, FEV1: forced expiratory volume in 1 s, IQR: inter quartile range.

The home and hospital spirometry measurements for the FEV1 (*r* = 0.99 (*p* < 0.01)) and FVC (*r* = 0.99 (*p* < 0.01)) were highly correlated (Pearson correlation coefficient). The mean difference between the home and hospital measurements was 0.09 ± 0.14 L for FEV1 and 0.19 ± 0.28 L for FVC, with generally lower readings for home spirometry. The median within-subject variability (coefficient of variation) was 5.3% for FEV1 (2.8–9.2%) and 5.9% for FVC (2.0–13.0%) (Figure 1). The variability for each individual patient is provided in Table S1. Two patients received follow-up training due to variable measurements at the beginning of the study.

The patient-reported adherence with the old PulmoLife spirometer at baseline was high; most patients (80%) answered that they performed daily spirometry, with only sporadic missing values. Two patients performed spirometry weekly, both stable patients many years after lung transplantation.

During the study, the adherence to weekly measurements was 100%. The median follow-up in the study was 93 (inter quartile range, 76–130) days.

### 3.3. Patient Experiences

**Figure 2:**
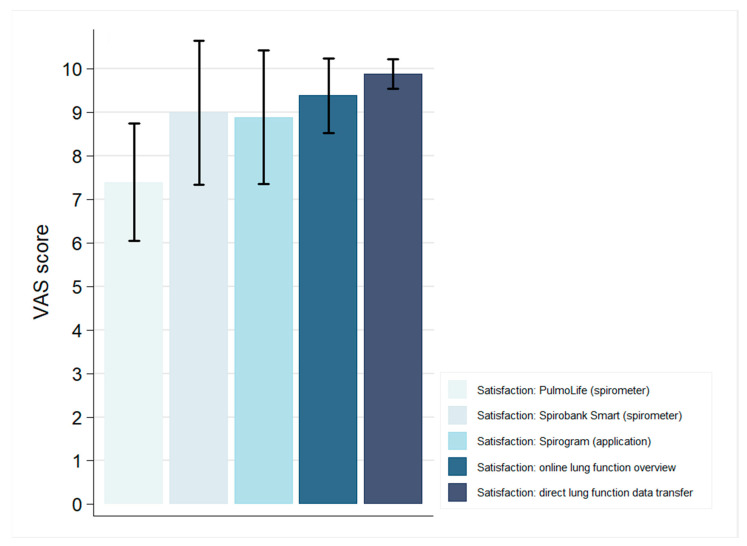
Mean patient satisfaction and experiences, scored on visual analogue scales from 0 to 10. Standard deviation (SD) is represented by the error bars. VAS: visual analogue scale.

At baseline, patient satisfaction (*n* = 10) with the PulmoLife Spirometer was high, with a mean VAS score of 7.4 ± 1.4 (Figure 2). Five patients reported occasionally doubting whether to contact the hospital. Two patients were often in doubt. For example, patients commented: “It remains difficult when to contact the hospital, you do not want to be a nuisance” and “I have experienced rejection once, I should have called earlier then”.

The vast majority (80%) of the patients answered that they would appreciate it if the lung transplant team monitored their lung function online; two patients did not have an opinion. Most patients (80%) would like to report physical symptoms in a home monitoring application, and four patients would like to track their step count.

At the end of the study, the patients evaluated the novel Spirobank Smart Spirometer. The patients (*n* = 9) considered the new online application (mean VAS score, 8.9 ± 1.5) and the Spirobank Smart Spirometer (mean score, 9.0 ± 1.7) easy to use. The patients highly appreciated the overview of their lung function over time (mean score, 9.4 ± 0.9) and the direct data transfer to healthcare providers (mean score, 9.9 ± 0.3) (Figure 2). All the patients preferred the use of the Spirobank Smart Spirometer over the PulmoLife Spirometer, with a difference of 1.4 ± 1.5 points (*p* = 0.02). Moreover, all the patients would like to continue using the online home monitoring application and would recommend it to others. Patient suggestions for the improvement of the application included, amongst other things, the possibility to add blood pressure, oxygen saturation, heart rate, glucose, weight, and temperature measurements.

None of the patients reported anxiety or depression at any time point, limiting the likelihood of finding any significant differences. No significant differences were found between the anxiety score (difference, 0.1 ± 1.7, *p* = 0.85) at baseline (HADS-anxiety mean score, 2.9 ± 2.0) and the end of the study (HADS-anxiety mean score, 2.8 ± 2.1). Likewise, no significant differences were found between the depression scores (difference, 0.4 ± 1.8, *p* = 0.48) at baseline (HADS-depression mean score, 1.3 ± 1.9) and the end of the study (HADS-depression mean score, 1.8 ± 0.8).

## 4. Discussion

This pilot study demonstrates that an online home monitoring application for the follow-up of patients after lung transplantation is feasible and reliable, and patient satisfaction is high. Home and hospital measurements of the FEV1 and FVC showed a very high correlation, providing a good alternative for hospital spirometry. The within-subject variability was low, and the adherence to weekly home spirometry was high.

The within-subject variability and good correspondence between the home and hospital spirometry in our study are in line with results from studies for other chronic lung diseases that used the same home monitoring application [9,13]. Moreover, the variability of the home spirometry measurements was comparable with a previous study with lung transplant recipients using a different system [8]. Hence, this novel home monitoring application seems suitable for use in daily practice and future studies.

In current daily practice, patients report their home spirometry results in a paper diary, which is discussed at the outpatient clinic. The patients have to contact the transplant center themselves when they experience lung function decline or increasing symptoms. Thus, non-adherence cannot be remotely controlled for, which might lead to the delayed detection of lung function decline. Furthermore, the quality of the measurements cannot be evaluated, and patients may be uncertain as to whether to contact the hospital.

Good adherence to home monitoring after lung transplantation is associated with better patient and graft survival; however, previous studies have shown that adherence decreases over time [4,6,14,15]. Although this could not be directly evaluated with our current study design, it is likely that patient adherence to home spirometry increases with the online monitoring of home measurements by the transplant team. Online home spirometry with direct data transfer could thus potentially lead to improved long-term patient and graft outcomes. Larger prospective studies with a longer follow-up should reveal whether this benefit can indeed be demonstrated.

In the present study, we took the first steps towards an online application developed together with lung transplant recipients. The patients evaluated a new online home monitoring application and provided suggestions for improvement to specifically tailor the application for lung transplant recipients. As also suggested by Geramita et al., an application specifically designed for, and adapted with input from, lung transplant recipients could possibly improve long-term adherence and enable personalized treatment for individual patients [14].

Moreover, by using an application with real-time data transfer as we did in the current study, non-adherence could be detected earlier. For example, the patient who was admitted with pneumosepsis did not measure lung function in the five days prior to admission due to already feeling ill, whilst earlier in the study, daily measurements were performed.

Studies in other chronic lung diseases with a similar application have also incorporated automated email alerts in the case of significant lung function decline [9]. When these automated alerts are integrated in the application for lung transplant recipients, lung function decline can potentially be easily detected, and patients who normally hesitate to communicate about lung function declines can be treated in a timely manner [4,15,16].

We hypothesized that the direct data transfer of the home spirometry with review by the lung transplant team could reduce anxiety, as previously, a home monitoring system providing standardized feedback regarding whether to contact the hospital was found to decrease anxiety levels [5]. Such an effect was not found in our study. However, none of the included patients experienced anxiety or depressive symptoms at baseline in contrast to in the study by Sengpiel et al. The higher initial anxiety and depression scores could be explained by the fact that in their study, patients were included directly after transplantation, whilst we included mostly stable patients longer after transplantation. It thus remains possible that the implementation of home spirometry with direct data transfer for all patients directly after transplantation could reduce anxiety.

Importantly, patient satisfaction with the home monitoring application was high, and all the patients preferred this novel application over the PulmoLife Spirometer. One patient reported technical difficulties, which emphasizes the need for good instruction and the availability of a technical helpdesk.

Additionally, a reliable online home monitoring system could facilitate novel forms of care, such as teleconsulting instead of real-life consultation. This is particularly relevant today, as due to the recent outbreak of SARS-CoV-2, many lung transplant recipients are hesitant to come to the hospital for consultation and lung function tests, and at the same time, patient traffic within hospitals has to be restricted [17].

A limitation of this study is that it was a single-center study, with a small sample size and a short duration. Nevertheless, we believe that this was sufficient for evaluating the feasibility, patient experiences, and suggestions for the improvement of the online home monitoring application. The patient suggestions will be used to further adapt and optimize the application for upcoming studies and integration in daily care, which will hopefully facilitate long-term use and improve adherence.

In conclusion, online home monitoring with direct data transfer is feasible and reliable after lung transplantation and results in high patient satisfaction. Larger prospective studies with a longer follow-up should reveal whether this online home monitoring application enables the earlier detection of lung function decline compared to current offline home monitoring, and thereby potentially improves the efficiency of care as well as patient and graft survival.

## Figures and Tables

**Table 1:** Baseline characteristics.

Variables		Patient (*n* = 10)	
Age	(Years (range))	67	(58–78)
Gender	Male	5	(50%)
	Female	5	(50%)
Time after transplantation	(Years (range))	8.04	(0.4–19)
Type of transplantation	Unilateral	1	(10%)
	Bilateral	9	(90%)
Underlying disease	COPD	3	(30%)
	A1AT deficiency-related emphysema	4	(40%)
	ILD	3	(30%)

COPD: chronic obstructive pulmonary disease; A1AT: alfa-1-antrypsin; ILD: interstitial lung disease.

## Supplementary Materials

The following are available online at https://www.mdpi.com/2075-4426/10/4/240/s1. Figure S1: Spirobank Smart Spirometer, Figure S2: Direct transfer of lung function data, Table S1: Coefficient of variation for each individual patient.
