# Supplementary material for: Evaluation of a Home Monitoring Application for Follow Up after Lung Transplantation—A Pilot Study
Source: J Pers Med. 2020 Nov 21;10(4):240. doi: 10.3390/jpm10040240 (PMC7711442; doi:10.3390/jpm10040240)
Supplement: Supplementary file 1 [file jpm-10-00240-s001.zip › Table S1 Coefficient of variation for each individual patient.docx]

| *Subject* | FEV1 | FVC |
| --- | --- | --- |
| *1* | 5.3 | 5.1 |
| *2* | 4.9 | 4.7 |
| *3* | 7.5 | 13.0 |
| *4^1^* | - | - |
| *5* | 3.1 | 5.0 |
| *6* | 9.2 | 6.2 |
| *7* | 2.8 | 2.0 |
| *8* | 5.8 | 8.0 |
| *9* | 7.4 | 6.0 |
| *10* | 3.0 | 3.1 |

Table S1: Coefficient of variation (%) for each individual patient.

^1^ Subject 4 was excluded from the analysis due to hospitalization.
