# Supplementary figures and images for: Evaluation of a Home Monitoring Application for Follow Up after Lung Transplantation—A Pilot Study
Source: J Pers Med. 2020 Nov 21;10(4):240. doi: 10.3390/jpm10040240 (PMC7711442; doi:10.3390/jpm10040240)

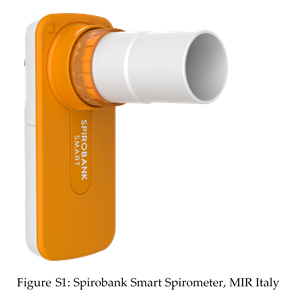

Supplement: Supplementary file 1 [file jpm-10-00240-s001.zip › Figure S1 Spirobank Smart.png]

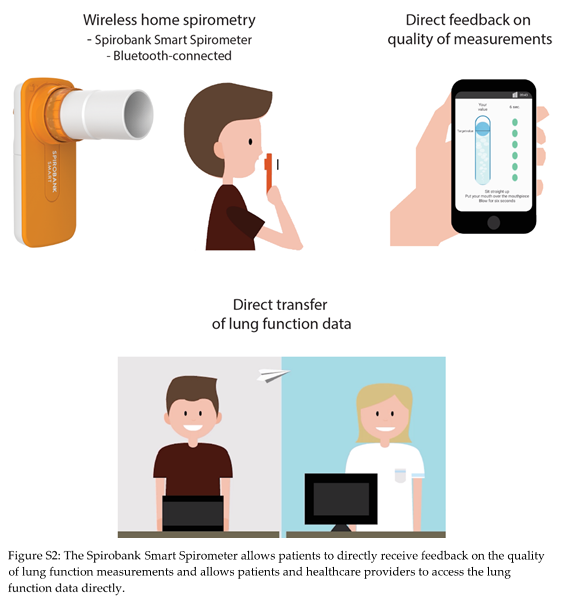

Supplement: Supplementary file 1 [file jpm-10-00240-s001.zip › Figure S2 Direct transfer of lung function data.png]
